# Being on the Field When the Game Is Still Under Way. The Financial Press and Stock Markets in Times of Crisis

**DOI:** 10.1371/journal.pone.0067721

**Published:** 2013-07-05

**Authors:** Roberto Casarin, Flaminio Squazzoni

**Affiliations:** 1 Department of Economics, University Ca’ Foscari, Venice, Italy; 2 Department of Economics and Business, University of Brescia, Brescia, Italy; Cinvestav-Merida, Mexico

## Abstract

This paper looks at the relationship between negative news and stock markets in times of global crisis, such as the 2008/2009 period. We analysed one year of front page banner headlines of three financial newspapers, the *Wall Street Journal*, *Financial Times*, and *Il Sole24ore* to examine the influence of bad news both on stock market volatility and dynamic correlation. Our results show that the press and markets influenced each other in generating market volatility and in particular, that the *Wall Street Journal* had a crucial effect both on the volatility and correlation between the US and foreign markets. We also found significant differences between newspapers in their interpretation of the crisis, with the *Financial Times* being significantly pessimistic even in phases of low market volatility. Our results confirm the reflexive nature of stock markets. When the situation is uncertain and unpredictable, market behaviour may even reflect qualitative, big picture, and subjective information such as streamers in a newspaper, whose economic and informative value is questionable.

## Introduction

Global financial crises have always followed similar patterns throughout history (e.g., [1]), including the crucial role of financial press (e.g., [2]; [3]; [4]; [5]). From the bursting of the Tulip buble in 1637 in the Netherlands to the dot.com bubble in 2001 in America, the financial press has significantly influenced the stock market, often amplifying the cognitive bias and herd behaviour of investors (e.g., [6]; [7]).

This behaviour may depend on the strong sensitivity of investors towards bad news. Recent studies have shown that market responses to good and bad news is asymmetric. Indeed, investors are more sensitive to negative news, especially when the market is dominated by uncertainty and unpredictability, and this is an important source of market volatility (e.g., [8]; [9]; [10]; [11]; [12]; [13]; [14]). [15] found that stock prices overreacted to bad news even in good times and underreacted to good news in bad times. Similarly [16] found that bad news has a bigger impact both in phases of market expansion and contraction. Moreover, the press may induce a “framing effect”, according to which investors react disproportionally to negative news especially when information source is authoritative (e.g., [17]; [18]; [19]). In this case, communication research indicates that the perceived authoritativeness of news sources implies higher trust in the news from these sources ([20]). This effect has been empirically confirmed by a survey on 321 traders and 63 financial journalists from leading banks and financial news providers in the European foreign exchange market ([21]) and a recent case-study on three Dutch banks during the recent financial crisis ([14]).

While the importance of these aspects has been largely underestimated in economics (e.g., [22], [23]), they have been recently investigated in empirical finance (e.g., [24]; [25]; [26]), with interesting parallels with recent sociological investigation on financial markets (e.g., [27]). Unlike the efficient market hypothesis, these studies show that investors are largely influenced by the media, rumours and gossip even in ‘normal’ market periods, where prices should contain all necessary information (e.g., [24]; [28]). If this is so, we would expect that, in times of financial crisis such as the 2008–2009 period, not only would the investors’ overreaction to bad news have drastically influenced market behaviour, it could even lead investors to overestimate the relevance of not strictly economic, general information. Indeed, in these situations, market prices and other relevant quantitative data on markets are even more variedly interpreted by investors than in normal periods ([27]). Even qualitative, big picture, subjective information, such as streamers in a newspaper, can become relevant in these cases.

To look at this, we investigated the relationship between negative news in financial newspapers and volatility and correlation between stock markets during the recent global financial crisis. We analysed one year of front page banner headlines of three financial newspapers, such as the Wall Street Journal, Financial Times, and Il Sole24ore from 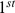 September 2008 to 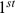 September 2009, when the recent financial crisis exploded globally. We created an index of bad news per newspaper on a daily base and studied the relation between this index and the closing values of three stock market indexes, such as the DowJones, FTSE and MIB. We considered these stock markets as they were more domestically affected by these newspapers, while comparing their dynamics was essential to look at equivalences and differences across markets and between different press cultures.

Our work has important differences compared with previous work. First, unlike previous studies in empirical finance (e.g., [25]), we focused on the last financial crisis as we wanted to better understand the fabric of pessimism that dominated the last years worldwide. Secondly, rather than considering specific market information, as reported in specialized columns, we looked at the impact of general information provided by front page headlines of the financial press. Indeed, front page headlines are crucial in summarizing the meaning, tone and importance of the news but are not expected to contain true, precise and detailed information about economic facts, unlike specialized columns. This is because: (i) headline information is too succinct and (ii) front page messages heavily reflect specific information strategies of the newspapers, which are mainly concerned with impressing and attracting the reader. Furthermore, unlike [24], we did not restrict our interest to strictly speaking financial news but considered economic information in general. Unlike [29] and [25], we did not focus on precise information concerned with specific stocks but rather looked at general information, which reflects more interpretation than objective details. Thirdly, while studies on the impact of social media on stock markets have recently been carried out that focus on similar crisis periods (e.g.,[30]; [31]), our idea was that, in a situation of financial turmoil, the authoritative columns of certain influential financial newspapers and so also traditional media could have a strong impact on the investors’ mood. Finally, by comparing three newspapers and their respective stock markets, we also wanted to measure differences in interpretation of this global crisis and consider certain country-specific cultural features of the press (e.g., [32]).

To our knowledge, this is the first work that extends the analysis of the impact of financial press from market volatility to market correlation. Combining volatility and correlation was key to: (i) understanding if bad news has had an effect on the growing interdependence of markets, which is presumably correlated with crisis periods (e.g., [9]; [33]); (ii) looking at the impact of bad news not only from the point of view of risk but also from that of risk diversification. We estimated the volatility and correlation dynamics using generalized autoregressive conditional heteroskedasticity (GARCH) models (see [34]) with dynamic conditional correlation ([35]). Secondly, we estimated the dynamic relationship between market volatility/correlation and bad news by using vector autoregressive models (VAR). We also performed a Granger-causality test to verify whether bad news time series had predictive value for market volatility/correlation ([36]). A strong quantitative approach to typical sociological and qualitative factors, such as investors’ mood and media pessimism, was intended to favour cross-fertilization between empirical finance (usually addressed to quantitative facts, but was little concerned with sociological aspects) and sociology of financial markets (strongly concerned with sociological aspects of markets, but poorly interested in macro quantitative market consequences). This integration is essential to understand complex institutional, socio-economic artefacts, such as financial markets.

The rest of the paper is organized as follows. Sect. 2 illustrates the background and our research hypotheses. Sect. 3 presents our dataset and illustrates the bad news index that we used to measure the relationship between newspapers and markets. It also shows data on market volatility and correlation. Sect. 4 introduces the model we built to examine the impact of newspapers on markets. Sect. 5 focuses on causal statistical relationships between the press and markets. Sect. 6 provides a robustness and sensitivity analysis, while the last Sect. summarizes our main findings and discusses certain limitations.

## Background and Hypotheses

Many studies in finance have shown that stock market prices incorporate financial press information (e.g., [37]; [38]; [39]). While this may be expected in cases of quantitative information on important economic statistics, such as those regularly released by important institutional agencies (e.g., [40]; [41]; [42]; [43], [44]; [45], [46]), it is less likely to find a positive impact of qualitative information, such as journalists’ opinion or reports of market rumours, which is subjective ([47]; [28]). Nevertheless, empirical evidence is also growing in this area.

For instance, [29] examined Dartboard, a monthly column of the Wall Street Journal reporting analysts’ recommendations, from 1988 to 1990. Results showed that for the two days following the publication, average positive abnormal returns of 4 percent of the stock recommended were partially reversed only within 25 trading days. Similarly, [25] examined Abreast of the Market, a popular column of the Wall Street Journal, from 1984 to 1999. It is worth noting that, unlike Dartboard, which asks market analysts’ opinions, this column is closer to entertainment than information. The author found that even qualitative information, such as the fraction of negative words in this column, was incorporated in aggregate market valuations. More specifically, results showed that high level of pessimism robustly predicted downward pressure on market prices and that high or low values of pessimism helped to predict high market trading volumes. More recently, [48] examined 30 years of “Abreast of the Market” and showed that even specific columnists can influence stock market behaviour. [49] extended this type of analysis by addressing the impact of negative words in all Wall Street Journal and Dow Jones News Service stories about individual S&P 500 firms from 1980 to 2004. Results showed that negative words in the financial press forecasted low firm earnings and that stock market prices incorporated the information embedded in negative words only with a slight delay. This would confirm that bad news is assimilated faster than good ones in market behaviour (e.g., [19]).

Other studies showed that this effect was even true for unconventional, un-specialized media, whose information should be less relevant for investors. For example, [24] examined the effect of more than 1.5 million messages posted on Yahoo! Finance and Raging Bull about 45 companies in the Dow Jones Industrial Average and the Dow Jones Internet Index, by measuring bullishness. They found that stock messages helped to predict market volatility both on a daily base and also within the same trading day. More specifically, they found that higher message postings predicted negative subsequent returns. They also found that disagreement between the posted messages was associated with increased trading volume. More recently, [31] found that Twitter mood predicted more than 80% of daily volatility of closing values of the Dow Jones Industrial Average. This would confirm Nofsinger’s argument that social mood may cause an increase of decisions biased by optimism or pessimism that could considerably influence aggregate investment and business activity, even reflecting future economic activities ([50]).

The idea that markets are influenced by reflexivity mechanisms has been explained by recent sociological investigation, which has mostly examined trading activities in specific organizational contexts (e.g., [51]; [23], [52]; [27]). Unfortunately, these studies underestimated the importance of understanding how context-specific empirical cases could result in aggregate quantitative market data. Our aim was to fill this gap by formulating and empirically testing certain hypotheses on the influence of press information on stock market behaviour comparatively, so as to consider possible institutional diversity in the relation between financial press and markets.

Our research hypotheses were as follows. First, [11] and [28] showed that investors, even those following long-term strategies, are more influenced by negative news as they reduce the difficulty in predicting future outcomes by overestimating the impact of current information (see also [9]; [12]; [13]; [16]). This was also found in experimental and economic psychology (e.g., [22]). Our hypothesis is that the importance of these psychological factors could dramatically increase during financial turmoil as investors tend to disqualify the reliability of prices and even the well-functioning of the markets and are more sensitive to other sources of information, including newspaper headlines. Indeed, the fact that information is subject to profit maximisation by newspapers should make investors cautious of these sources ([53]). Furthermore, unlike efficient market hypothesis, experimental research has shown that, in uncertainty, investors tend to overestimate their informational gap and are sensitive to any additional information, including subjective one (e.g.,[54]). Therefore, our first hypothesis (H1) is that bad news published by financial newspapers could negatively influence the daily volatility of financial markets during this period.

Secondly, although most financial crises have had an international impact in the past, the 2008–2009 crisis was truly global as financial markets are now extremely interdependent. Indeed, modern investment technologies allow investors to make millions of operations per time unit, at any time and anywhere (e.g., [55]). In this situation, we expect that the pessimistic messages of financial newspapers could explain not only market volatility but also dependence between stock markets. Numerous previous studies showed that market interdependence tends to be highly correlated with periods of volatility (e.g., [56]; [22]). For instance, [9] and [33] suggested that in periods of crisis and high market volatility, covariation could even include markets that do not have much in common. [57] suggested that this trend has intensified especially recently with increasing globalization of investment strategies. Our hypothesis (H2) is that in periods of turmoil, bad news could even influence market correlation and has an impact on global investment strategies. Coherently, we expected that in this period the interplay of financial markets and the press could determine a cascade of pessimism that co-influences both information and market behaviour (H3) (e.g., [58]).

## Data

### The Bad News Index

Our dataset includes one year of front page banner headlines of three financial newspapers, namely the Wall Street Journal, Financial Times and Il Sole24ore, on a daily base. For technical constraints, i.e., the unavailability of fully accessible electronic editions or lack of front page news included in electronic versions, we collected data manually on printed versions of the newspapers. Budget, time and linguistic constraints did not permit us to consider other national newspapers or cover longer time periods. The dataset and a text file, including a full description of the variables, are available as supplementary information. We analysed any front page banner headline from 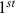 September 2008 to 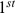 September 2009 which conveyed news on the crisis (not only those expressly related to financial markets) by measuring the emphasis and the tone of the message. The emphasis was measured by counting the number of banner columns reporting an economic news compared with the total number of potentially available columns, according to standard newspaper layout. We assumed that the higher the percentage of columns assigned to the banner headline, the stronger the emphasis of the message was. The tone was measured by counting the ratio of negative words over the total words used in the headline text (all included, also verbs and conjunctions), such as “recession”, “fear” and so on. We assumed that the higher was the number of negative words in the text, the stronger the pessimism of the message was. For the sake of simplicity, we did not distinguish the degree of pessimism by raking the words used.

Our bad news index was based on three types of information. We considered the number of negative banner headlines on the crisis, 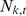, the number of columns, 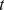, where news were reported, and the number of negative words reported in the text, 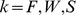, at time 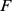 for each journal 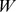, where 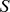 stands for Financial Times, 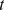 for Wall Street Journal, and 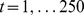 for IlSole24ore. It is worth noting that the time index 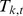 refers to open-trading days, so time 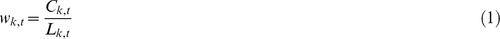 days. The information from the press during a non-trading day is reported together with the information of the first subsequent open-trading day. The index was build as follows. Let 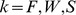 be the maximum number of available columns for a banner headline in the newspaper, then the relative importance index was
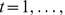
(1)

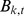
 and 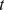. Then the journal-specific bad news index, 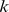, at time 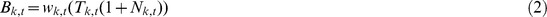 for the journal 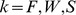, was defined as
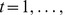
(2)for 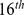 and 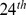.

### Descriptive Statistics

**Figure 1:**
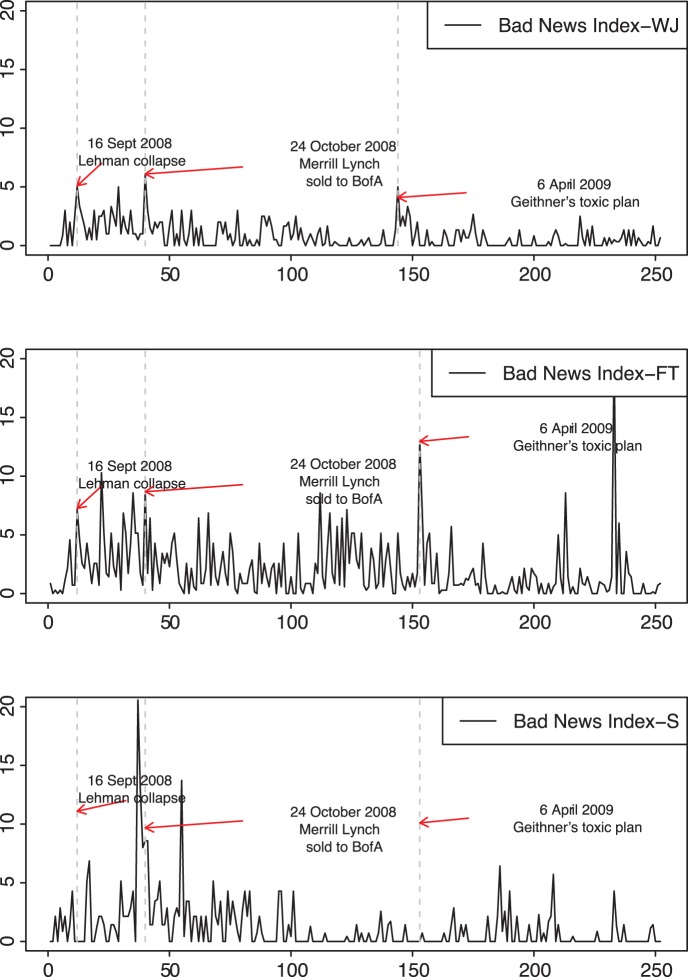
Bad news index per newspaper (in rows) from 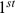 September 2008 to 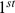 September 2009 on a daily base. Peaks of bad news are indicated with vertical dashed lines.

Figure 1 shows the bad new index per newspaper. The vertical dashed lines correspond to certain peaks of bad news. The first peak was on 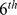 September 2008, the day before it was announced that Lehman Brothers filed for Chapter 11 bankruptcy protection, Merrill Lynch agreed to be sold to Bank of America for 50 billion dollars and estimates said that up to 50.000 jobs were at risk. The second peak was on 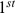 October 2008 after the Congressional hearing where Alan Greenspan admitted that he had put too much faith in the self-correcting power of markets. A third peak was on 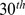 April 2009, involving especially the Financial Times, when the Geithner plan to buy toxic assets was strongly criticised as a means to provide government “cash for trash” and UK analysts started to forecast that stagflation was around the corner.

If we consider bad news dynamics, our one-year sample could be approximately divided into two sub-samples, i.e., an initial period characterized by higher concentration of bad news, and a second one after spring 2009, where bad news were generally less frequent. By comparing the three newspapers, it is evident that the Wall Street Journal was more cautious and the Financial Times published bad news more frequently, also for the second sub-sample.

Tab. 1 shows mean, standard deviation, skewness and kurtosis of the bad news index per newspaper. Looking at the mean value of the bad index, it is worth noting that the Financial Times was more pessimistic than the other newspapers. On the other hand, if we consider the deviation from the mean (skewness) and the extreme values (kurtosis), Il Sole24Ore showed higher excess pessimism. The Wall Street Journal was more cautious throughout the entire sample, i.e., it showed both lower means and volatility.

**Table 1:** Descriptive statistics of the bad news index.

	Whole Sample				First Sub-Sample				Second Sub-Sample			
	Mean	St.D.	Sk.	Kurt.	Mean	St.D.	Sk.	Kurt.	Mean	St.D.	Sk.	Kurt.
W	0.82	1.07	1.69	6.41	1.09	1.22	1.30	4.74	0.42	0.61	1.47	4.64
F	1.98	2.49	2.73	15.86	2.38	2.22	1.10	3.67	1.40	2.78	4.39	26.79
S	1.17	2.31	4.12	27.41	1.60	2.76	3.56	20.33	0.55	1.19	2.84	11.85

	Whole Sample			First Sub-Sample			Second Sub-Sample		
	**W**	**F**	**S**	**W**	**F**	**S**	**W**	**F**	**S**
W	1.00*	0.19*	0.13*	1.00*	0.20*	0.08	1.00*	0.03	−0.06
F		1.00*	0.11*		1.00*	0.06		1.00*	0.13
S			1.00*			1.00*			1.00*

First panel: mean, standard deviation, skewness and kurtosis. Second panel: correlation between indexes. The symbol “*” indicates that the null hypothesis of zero valued Pearson’s correlation was rejected at the 5% significance level.

We then distinguished two sub-samples, the first from 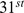 September 2008 to 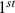 March 2009, where the market volatility was considerably higher, the second from 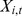 March 2009 to 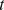 September 2009, with less volatility. Data showed that pessimism was generally higher in the first sub-sample. The Financial Times and Wall Street Journal showed a similar level of excess pessimism, which was lower than Il Sole24Ore. In the second sub-sample, where market volatility was lower, the Financial Times showed both higher levels of pessimism and excess pessimism. This meant that the Financial Times followed a more critical stance on the crisis, by reporting bad news even in periods of relatively lower market volatility.

Our database also included the names and amount of journalists who authored any front page leading article on the crisis. We calculated a Gini index that measured the concentration of articles per journalist. Not only did the Financial Times concentrate more articles with a few journalists (WSJ index took 0.61, while FT took 0.66), it did so especially in the low market volatility period (WSJ index for the second sub-sample took 0.68, while FT took 0.77). This meant that the stronger critical stance of Financial Times in times of lower market volatility was due to a few critical journalists (see more detailed analysis in Sect. 6).

These findings can be explained by considering certain historical and institutional differences between the U.S. and UK financial press (e.g., [59]). [60] has argued that the higher pessimism of the Financial Times in reflecting the 2008/2009 crisis depended on a mixture of history and contingency (see also [61]). On the one hand, while the Wall Street Journal has been historically more devoted to investigation, addressed to an investor readership and focused on domestic affairs, the Financial Times has always been more concerned with economic theory and interpretation and mainly focused on international affairs. This could explain the stronger sensitivity of the British newspaper towards a general outlook of the crisis (e.g., the implications of the financial crisis for the real economy) and its stronger focus on commentaries and academic debate. Even the tendency to blame U.S. market responsibility and critically discuss the U.S. political agenda against the crisis could explain the stronger sensitivity of the Financial Times towards the development of the crisis (see also [62]). It is worth noting that more than 80% of Financial Times front pages in the period considered included an article or commentary on the crisis, against 67% of Wall Street Journal.

On the other hand, the stronger concern for home investors could have lead the Wall Street Journal to follow a less critical stance and be more cautious in spreading bad news. In a recent story on the U.S. press coverage of the financial crisis, [63] suggested that American journalists were extremely cautious in reporting bad news as it was clear that, in a situation of market unpredictability and turmoil, any influential opinion or streamer could have had a dramatic influence on market behaviour.

Secondly, it is worth noting that after the dramatic events of September/October 2008, the financial press in the UK was strongly criticized for boosterism and excessive embeddedness. [60] explained that, in the autumn of 2008, a turning point was achieved in the relationship between press and markets, epitomized especially by the Financial Times. This was called the “media’s moral compass” to mean that the relationship between the press and the market shifted from a “cozy co-dependence” to a more critical stance. This would explain why the Financial Times suggested a pessimistic interpretation of economic events.

The next step was to calculate the correlation between newspapers (see Tab. 1). Results showed that newspaper pessimism was significantly positively correlated. The more positive correlations were between the Wall Street Journal and Financial Times, and between the Wall Street Journal and Il Sole24ore. If we consider the difference between periods of high and low market volatility, it is worth noting that the higher correlation was in high volatility periods between Wall Street Journal and Financial Times, whereas correlations were not-significant or negative in periods of low volatility. This meant that in periods of higher market volatility, differences between Wall Street Journal and Financial Times drastically diminished. Indeed, in this period, these two leading newspapers basically conformed both in terms of timing and intensity of pessimism. On the other hand, significant differences persisted for other periods, where market volatility was less pronounced.

### Market Volatility and Correlation

Let 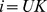 indicate the log-return at time 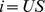 for FTSE (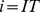), DowJones (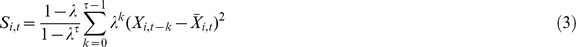) and MIB (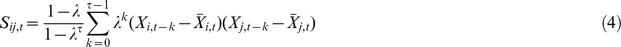), the three stock market financial indexes to which the three newspapers refer more frequently. Let us calculate their volatility and correlation dynamics by means of a generalized autoregressive conditional heteroskedasticity (GARCH ) model (see [34]; [64]) with dynamic conditional correlation (see also [35]). For the sake of simplicity, we followed a non-parametric approach as follows:
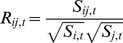
(3)

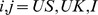
(4)

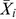
(5)for 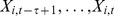 where 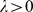 was the empirical average over 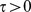, 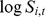 was a smoothing factor and 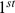 was a forgetting factor.

**Figure 2:**
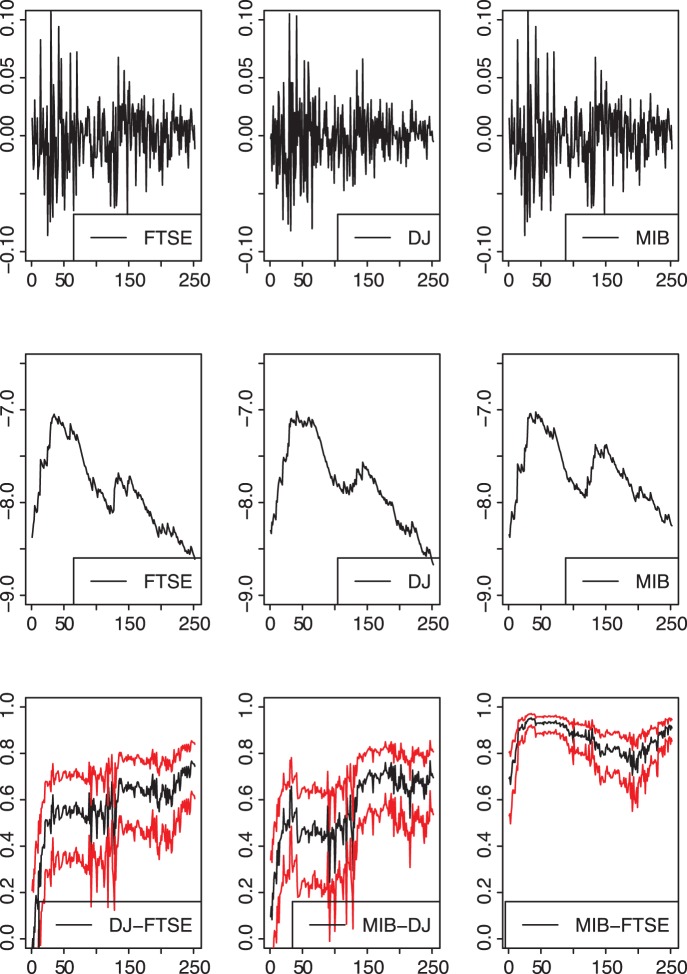
Daily log-returns (first row) of the FTSE, DowJones and MIB indexes from 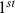 September 2008 to 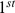 September 2009. Daily log-volatilities (second row) and correlations (third row), evaluated sequentially over time with a rolling window of 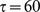 observations and a smoothing factor 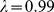. In the last row, the red lines indicate the 95% confidence band about the estimated correlations.

Fig. 2 shows the results of our non-parametric estimation procedure. The first row shows the log-returns of the FTSE, DowJones and MIB market indexes at a daily base for closing values. The graphs in the second row show the level of log-volatility (i.e. 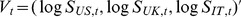) for each index. Although we did not report data before 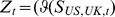 September 2008, it is worth noting that market volatility significantly increased after September 2008. This is evident when looking at the beginning of our sample (see the graphs in the second row of Fig. 2). It is worth clarifying that this was not due to a lack of data in the estimates at the beginning of the sample. Indeed, the first estimate of the volatility was calculated starting from a window of 60 initial observations, which were not represented in the first row of Fig. 2. This means that our sample fully reflected a period of significant market turmoil. Results showed that market dynamics were similar. More specifically, while the level of volatility was similar at the beginning, at the end of the sample the Italian market showed higher log-volatility than the UK and U.S. stock markets. Furthermore, the level of correlation between the three market indexes increased after September 2008 to quickly reach 0.6 for DowJones and FTSE, 0.7 for MIB and DowJones and 0.9 for MIB and FTSE. Secondly, our results showed that the correlation between these three markets was positive. If we look at values before and after the beginning of the period under observation, it is worth noting that the UK and Italian markets were more highly dependent than the U.S. and UK and U.S. and Italy respectively.

These results confirm certain previous empirical findings on the higher correlation between markets during a financial crisis (e.g., [65]; [66]), especially in periods of higher volatility of the U.S. stock markets ([67]). Secondly, they would confirm recent findings on the increasing interdependence of European stock markets ([68]).

### Analysis

Let 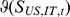 be the vector of log-volatilities and 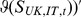, 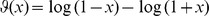, 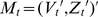, with 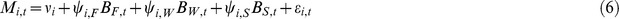, the vector of logistic-transformed correlations. Let us define 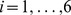. We examined the relationship between bad news and the variance and correlation of the three financial indexes. We considered static models as follows:
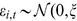
(6)with 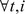, and 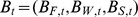 i.i.d. 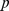.

Table 2 shows that all bad news coefficients were positive. This means the any increase of pessimism by newspapers had a positive impact on the volatility of markets. Obviously, the impact was not the same for each newspaper or market. The Wall Street Journal had a strong impact on all markets. The bad news of Wall Street Journal and Financial Times had a significant impact (at 5% significance) on the simultaneous level of log-volatility in all markets (see the left panel in Tab. 2). Finally, Il Sole24Ore bad news influenced the volatility of the UK and Italian stock markets.

**Table 2:** Left: the effect of the bad news indexes on volatility; Right: the effect of the bad news indexes on correlations.

Impact on volatilities				
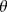	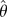	t-stat	p-val	
US				
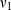	−7.6856	−192.1191	0.0000	*
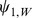	0.1332	4.6755	0.0001	*
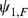	0.0266	2.8681	0.0044	*
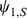	0.0354	2.8001	0.0551	
UK				
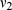	−7.7634	−202.5992	0.0000	*
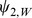	0.1198	5.0512	0.0001	*
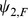	0.0323	3.1772	0.0017	*
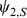	0.0395	3.2643	0.0012	*
IT				
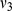	−7.4971	−236.6942	0.0000	*
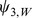	0.0731	3.7321	0.0002	*
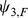	0.0234	2.7753	0.0059	*
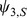	0.0301	2.9962	0.0031	*
**Impact on correlations**				
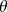	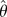	**t-stat**	**p-val**	
US-IT				
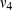	0.5818	39.6552	0.0000	*
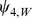	−0.0161	−1.7674	0.0119	*
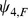	−0.0011	−0.2851	0.7762	
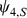	−0.0028	−0.5983	0.5502	
UK-IT				
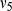	0.8334	122.7822	0.0000	
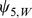	0.0108	2.5831	0.0104	*
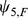	0.0037	2.0676	0.0398	*
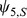	0.0048	2.0397	0.0225	*
UK-US				
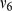	0.5862	38.0473	0.0000	*
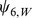	−0.0206	−2.1631	0.0212	*
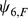	0.0021	0.5152	0.6068	
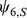	−0.0020	−0.4141	0.6790	

Columns: the parameter 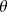 (first), estimates 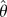 (second), value of the t-statistics (third), p-value of the t-statistics (fourth) and :*” indicates significance of the parameter at the 5% significance level (last).

As regards to market correlation, it is worth noting that Wall Street Journal’s pessimism had a significant impact on all correlations. Vice-versa, the Financial Times and Il Sole24Ore had an impact only on their respective markets (see the right panel of Tab. 2). This would confirm the leadership of Wall Street Journal in influencing the stock market and its worldwide impact. On the other hand, it is interesting to note that any increase of pessimism by the Wall Street Journal had a negative impact on the correlation between UK and Italian markets. This could be explained by the fact that the Wall Street Journal mainly focused on domestic affairs and negative news on the U.S. stock market could have lead investors to move their investments towards other markets or in general to explore a variety of investment strategies. This could have contributed to generate heterogeneity in stock market behaviour globally.

## Statistical Causal Relations

To look at the causes of statistical relationships in more detail, we performed a Granger-causality test that examined the lagged dependence structure between bad news and market correlation and volatility. This allowed us to verify whether bad news had any predictive value for market volatility and correlation. We considered each possible dependence between markets and information, by setting 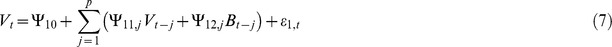 and considering VAR models.

### Volatility

A joint test on the statistical causal relationships of volatility and pessimism was based on the following VAR model of dimension 6 and order 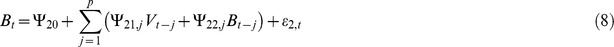:
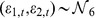
(7)

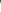
(8)with 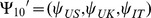 i.i.d. 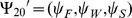, 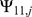 and 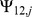 were the intercept and the 3-dimensional square matrices 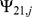, 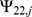, 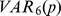, 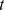, were the autoregressive coefficients of the 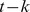 model.

In order to disentangle the relation between volatility and the press, we first looked at the statistical significance of the relationship between volatility at time 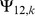 and news at time 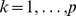, which depended on the matrices 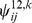, 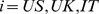 with elements 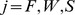, 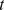 and 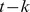. Then, we looked at the relation between bad news at time 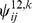 and volatility at time 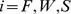, which depended on elements 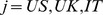, 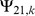 and 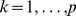 of the matrices 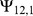, 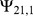. For our purpose and for the sake of simplification, we have included only a subset of the VAR coefficients, i.e., the elements of 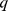 and 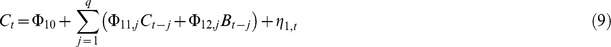 only for the first lag (see Table 3).

**Table 3:** Left: the effect of the bad news indexes at the first lag on volatility.

Impact on volatilities				
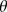	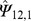	t-stat	p-val	
US				
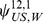	0.0093	3.1230	0.0020	*
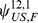	0.0002	0.1692	0.8663	
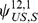	−0.0033	−2.3871	0.0177	*
UK				
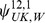	0.0076	2.2512	0.0253	*
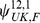	0.0002	0.1679	0.8676	
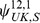	−0.0019	−1.1534	0.2501	
IT				
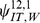	0.0073	2.1151	0.0354	*
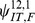	0.0004	0.3061	0.7602	
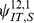	−0.0023	−1.455 0	0.1469	
**Impact on newspapers**				
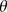		**t-stat**	**p-val**	
W				
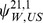	−0.32445	−0.4851	0.6278	
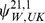	1.70194	2.4692	0.0142	*
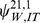	−0.96905	−1.8552	0.0648	
F				
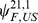	−2.0504	−1.2334	0.2188	
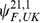	3.56990	2.0822	0.0384	*
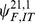	0.00951	0.0072	0.9941	
S				
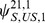	−2.9694	−2.1252	0.03462	*
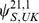	4.58829	3.1842	0.00164	*
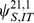	0.2066	0.1892	0.8501	

Right: the effect of volatility at the first lag on the bad new indexes. Columns: the parameter 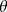 (first), estimates 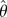 (second), value of the t-statistics (third), p-value of the t-statistics (fourth) and “*” indicates significance of the parameter at the 5% significance level (last).

Results showed that Wall Street Journal bad news (lagged by one period) significantly increased the volatility of the three market indexes in the subsequent period (see the left panel in Tab. 3). This is further confirmation of the leadership of this newspaper and its worldwide impact. The bad news of other newspapers did not have any significant impact on market volatility, with the exception of the lagged relation between Il Sole24Ore bad news and the volatility of the U.S. market. Considering the effect of market volatility on bad news, it is worth noting that the FTSE volatility had a significant and positive impact on the bad news of each newspaper (see the right panel in Tab. 3). This would confirm the recent worldwide importance of the London stock market. Finally, it is worth noting that the volatility of the Dow Jones index had a significant and negative impact on Il Sole24ore bad news.

We tested the hypothesis that volatility did not jointly statistically cause, in a Granger sense, the bad-news indexes. To look at the causal relationship between each market-specific volatility and the three newspapers, we also tested separately the hypothesis that neither each one of the three bad-news indexes, nor all three indexes jointly considered statistically caused, in a Granger sense, the market-specific volatility. To look at the relationship between each newspaper and the three markets, we did the same for newspaper bad news.

Tab. 4 shows the results of these joint and pairwise tests. First, the p-value of the joint test in the last column and last row, in the left panel, indicates that newspaper bad news was fully caused by market turmoil. Therefore, stock market behaviour was the essential source of bad news and newspapers did not have unrealistically pessimistic stances. Secondly, if we look at the p-values of almost all the pairwise causality tests (see the left panel), this statistical causality direction from markets to newspapers was true for all log-volatility and bad news indexes. On the other hand, if we look at the p-values in the last column, last row in the right panel, we should conclude that, in general, the financial press did not determine market volatility. More specifically, results showed that Wall Street Journal bad news alone had a strong statistical, causal impact on market volatility (see the first row in the right panel). Generally speaking, we could not predict market volatility only by looking at financial press bad news.

**Table 4:** Pairwise and joint causality test p-values.

	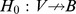				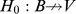			
	US	UK	IT	All	US	UK	IT	All
W	0.0012*	0.0036*	0.0426	0.0011*	0.0000*	0.0248*	0.0086*	0.0100*
F	0.0042*	0.0000*	0.0010*	0.0024*	0.1289	0.9817	0.8260	0.7534
S	0.0009*	0.0001*	0.0008*	0.0010*	0.4934	0.7773	0.8918	0.7251
All	0.0000*	0.0000*	0.0000*	0.0000*	0.7442	0.7573	0.7536	0.2550

The null hypotheses (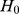) were as follows: volatility (V) did not cause (in the Granger sense) financial press pessimism (B) (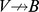, left panel), financial press did not cause volatility (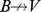, right panel). “All” indicates all variables included in the test and “*” indicates that the null is rejected at the 5% significance level.

### Correlations

The VAR model of order 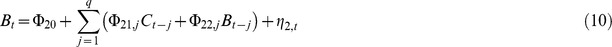 for bad-news indexes and correlations was as follows:
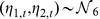
(9)

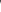
(10)with 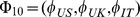 i.i.d. 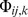, where 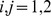 and were the intercept and 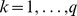, 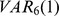 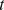, were the autoregressive coefficients of the 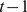 model.

We examined the statistical significance of the relationship between correlation at time 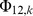 and bad news at time 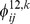, which was given by the matrix 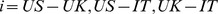 with elements 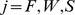, 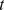 and 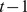. Then, we also looked at the relationship between bad news at time 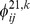 and correlation at time 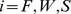, which was given by the elements 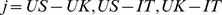, 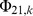 and 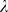 of the matrix 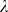. Note that, for the shortage of space, we included only the autoregressive coefficients at the first lag, not all the estimated coefficients of the VAR models (see Table 5).

**Table 5:** Left: the effect of the bad news indexes at the first lag on market correlations; Right: the effect of market correlations at the first lag on the bad new indexes.

Impact on correlations				
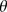	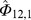	t-stat	p-val	
US-UK				
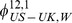	−0.0023	−2.3541	0.0193	*
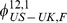	0.0004	0.4882	0.6263	
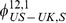	0.0016	0.7422	0.4589	
US-IT				
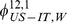	0.0005	0.2482	0.8044	
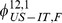	−0.0003	−0.2901	0.7718	
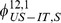	−0.0029	−2.7522	0.0064	*
UK-IT				
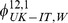	0.0001	0.1411	0.8881	
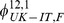	0.0004	1.1182	0.2646	
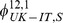	−0.0005	−1.1302	0.2598	
Impact on newspaper				
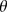	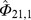	t-stat	p-val	
W				
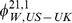	−4.6537	−3.5851	0.0004	*
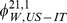	3.4669	2.8091	0.0053	*
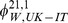	5.9194	4.0342	0.0000	*
F				
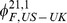	−2.3772	−0.7242	0.4700	
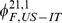	1.6567	0.5301	0.5962	
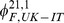	7.4877	2.0162	0.0449	*
S				
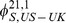	−8.0680	−2.9283	0.0037	*
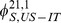	5.1940	1.9836	0.0485	*
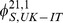	12.8542	4.1272	0.0000	*

Columns: the parameter 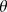 (first), estimates 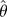 (second), value of the t-statistics (third), p-value of the t-statistics (fourth) and “*” indicates the significance of the parameter at the 5% significance level (last).

Our results (see Table 5, left column) showed that Wall Street Journal bad news (one lag) had a negative impact on the correlation between U.S. and UK stock markets. Indeed, bad news in the Wall Street Journal reduced the co-movement of these markets. This could be explained in terms of outflow of capital from the U.S. stock market and inflow into the UK market. On the other hand, Financial Times bad news (one lag) had no significant impact on market correlations. Il Sole24ore bad news decreased the correlation between the U.S. and Italian stock markets. The same explanation is a possible reason.

In addition, results (see Table 6, right column) showed that Wall Street Journal bad news reflected all one lag correlations. An increase in the correlation between the U.S. and UK stock markets decreased the journal’s pessimism, whereas an increase in the U.S.-IT and UK-IT correlations increased it. Furthermore, higher (one lag) correlation between the UK and Italian stock markets increased the pessimism of the Financial Times. Finally, all correlations had a significant impact on Il Sole24Ore, similar to the Wall Street Journal.

**Table 6:** Pairwise and joint causality test p-values.

	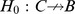				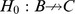			
	US-IT	UK-IT	US-UK	All	US-IT	UK-IT	US-UK	All
W	0.1017	0.0295*	0.0595	0.0001*	0.0121*	0.6951	0.0451*	0.0221*
F	0.6570	0.0100*	0.0108*	0.0223*	0.4739	0.3627	0.3627	0.6034
S	0.1026	0.0029*	0.0029*	0.0001*	0.9723	0.8733	0.8733	0.4141
All	0.1455	0.0026*	0.1780	0.0066*	0.0219*	0.4631	0.0208*	0.0423*

The null hypotheses (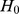) were as follows: correlation (C) did not cause (in the Granger sense) financial press pessimism (B) (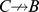, left panel), financial press did not cause correlation (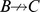, right panel). “All” indicates all variables are included in the test and “*” indicates the null is rejected at the 5% significance level.

We performed a joint and pairwise Granger causality test to rigorously asses the presence of causal relationships, as we did for log-volatility. The last column, last row in the left panel of tab. 6 shows that all correlations had a Granger causal effect on all bad news indexes. More specifically, while U.S.-IT stock market correlation did not statistically cause the bad news index, UK-IT and U.S.-UK correlations had a Granger causal effect on the bad news indexes of Financial Times and Il Sole24ore. Secondly, looking at the last column, last row of the right panel, we can see that the joint test did not reject the null hypothesis of the absence of Granger causality between all bad news and correlation indexes. Finally, results showed that Wall Street Journal bad news determined U.S.-IT and U.S.-UK market correlation but not that of UK-IT.

Therefore, any bad news in the Wall Street Journal predicted correlation between the U.S. and the other stock markets. Following certain peculiarities of Wall Street Journal as discussed in Sect 3 (see Table 2), such as its worldwide recognized leadership and strong focus on domestic affairs, this meant that investors considered any bad news published in this influential newspaper as a good prediction of market behaviour and promptly reacted by drastically modifying their global investment strategies.

To sum up, we can say that results corroborated the first hypothesis (H1), i.e., bad news published in the newspapers’ banner headlines had a significant influence on market volatility. More specifically, we found that Wall Street Journal alone contributed to market volatility. At the same time, our findings also corroborated the second hypothesis (H2), which argued that bad news could even influence the correlation of markets. More specifically, we found that the Wall Street Journal had a significant impact on market correlation, although different for different markets involved and directions. H3 was not fully confirmed as it claimed that, in period of financial turmoil, the press and markets influenced each other, possibly contributing to a cascade of pessimism. We found that market correlation and newspapers exerted mutual influence only in specific cases. In particular, our results showed that the Wall Street Journal strongly predicted market correlation and volatility.

Our findings confirm the sociological argument of the reflexive nature of stock markets. We found that media and markets are so systematically related to extend this reflexivity also to qualitative, subjective, broad picture information sources, whose true economic value should be seriously questioned from a mainstream, ‘efficient market hypothesis’ approach. On the other hand, especially if we look at the stronger influence of Wall Street Journal, we can argue against the common sense belief that newspapers would have over-exaggerated the dramatic events of 2008/2009 by imposing a critical stance which contributed to a cascade and contagion of pessimism.

## Robustness and Sensitivity Analysis

In order to corroborate our findings better, we performed further statistical tests to verify if certain specificities of market behaviour, such as downturn or upturn phases, could have had a consequence on the predictive power of the bad news index on market volatility and correlation. To do so, we re-estimated the VAR models for volatility and correlation described in the previous section by using alternatively the lowest and the highest returns of each trading day in the calculation of volatility and correlation. This was to estimate the impact of bad news on more point-to-point market behaviour, where it is less likely that stock markets fully reverberated all potential effects of a bad news.

Table 7 shows that Wall Street Journal bad news had a significant impact on extreme returns (see High and Low columns), so confirming previous findings. Furthermore, results showed that the Wall Street Journal not only had a significant effect on the U.S.-UK market correlation, but also on other correlations. On the other hand, the effect of market correlation on the Wall Street Journal bad news was more significant for closing returns than for low and high returns. This meant that the higher the correlation between markets, especially between the U.S. and UK markets, the lower the bad news index of the Wall Street Journal was. The situation with the Financial Times was different. In this case, the effect of correlation for low and high returns were statistically stronger than that of closing returns. Il Sole24ore showed weak correlation effects in these downturn and upturn cases. It is important to note that the FTSE-MIB correlation had a strong effect on the Il Sole24ore bad news independent of the type of market phases.

**Table 7:** Robustness analysis of the dynamic analysis for close, high and low returns.

Impact on correlations						
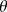	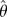					
	Close		High		Low	
US-UK						
	−0.0023	*	−0.0047	*	−0.0056	*
	0.0004		−0.0009		0.0006	
	0.0016		0.001		0.0005	
US-IT						
	0.0005		−0.0061	*	−0.0072	*
	−0.0003		−0.0003		0.0007	
	−0.0029	*	−0.0003		0.0003	
UK-IT						
	0.0001		0.0121	*	0.0118	*
	0.0004		0.0006		0.0008	
	−0.0005		−0.0018		−0.0024	

We then examined the robustness of the results on the choice of the smoothing 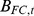 factor in the estimation of the volatility and correlation. Lower values of 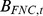 corresponded to higher weights for the most recent observations in the window considered. This leads to volatility and correlation that were more sensitive to large variations in returns. Thus, we verified whether these results survived to the inclusion of a higher level of noise in the estimation of variance and correlation. Table 8 shows that Wall Street Journal bad news index was significant for the DJ-FTSE correlation as in previous analysis, but that it was also significant for DJ-MIB correlation. It is worth noting that, in this case, the Financial Times became statistically significant for FTSE-MIB correlation.

**Table 8:** Sensitivity analysis of the impact of different choices of the smoothing factor on volatility and correlation estimations.

Impact on correlations						
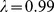	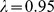					
	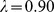		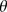		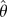	
US-UK						
	−0.0023	*	−0.015	*	−0.0242	*
	0.0004		0.0013		0.0032	
	0.0016		−0.0003		−0.0022	
US-IT						
	0.0005		−0.0175	*	−0.0256	*
	−0.0003		0.001		0.0019	
	−0.0029	*	−0.0021		−0.0039	
UK-IT						
	0.0001		−0.0003		0.0001	
	0.0004		0.0026	*	0.0047	*
	−0.0005		0.0017		0.0034	

We also investigated the case of the Financial Times better, trying to understand especially whether the relationship between its bad news and stock market was influenced by a small number of critical journalists. First, we distinguished two bad news indexes, the first, 
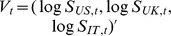
, for the group of central journalists and the second, 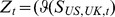, for the residual group (non-central journalists). We assumed that a journalist was central when he/she wrote more than nine commentaries in periods of high newspaper’s pessimism. We defined a period of high pessimism whenever the Financial times bad news index was higher than two. These criteria gave us nine central Financial Times journalists, such as Krishna Guha, Francesco Guerrera (now at the Wall Street Journal), and Ralph Atkins among others.

**Figure 3:**
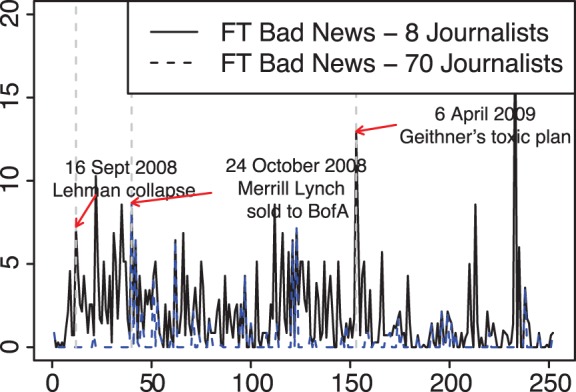
*Financial Times* bad news index per group of journalists from 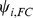 September 2008 to 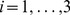 September 2009 on a daily base: central journalists are in black solid lines, non-central journalists are in blue dashed lines. Peaks of bad news are indicated with vertical dashed lines.

Fig. 3 shows that the two indexes for the two groups had different dynamics and often moved in opposite directions leading to a negative correlation −0.2305 (statistically significant at the 5% level). We then considered 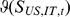 and 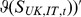, 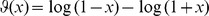, 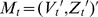, with 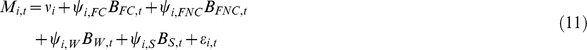 and 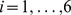 as in the previous sections and estimated.
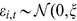
(11)with 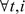, and 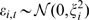 i.i.d. 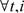.

Table 9 shows that the bad news index of the group of central journalists had a significant impact (at the 5% significance level) on the contemporaneous level of log-volatility in all markets (see the left panel). On the contrary, there was no relationship between the pessimism of non-central journalists and market volatility and correlation. This could indicate that the opinion of certain influential commentators was more considered by the market and revealed a specific strategy of the newspaper, i.e., to assign commentaries to more critical journalists in specific phases of the crisis.

**Table 9:** Left: the effect of the Financial Times central and non-central journalists (parameter 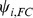, 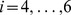) on volatility; Right: the effect of the Financial Times central and non-central journalists (parameter 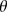, 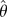) on correlations.

Impact on volatilities				
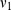	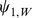	t-stat	p-val	
US				
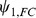	−7.1128	−169.9311	0.0000	*
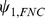	0.1284	4.7021	0.0001	*
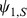	0.0266	3.1034	0.0021	*
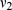	0.0266	1.4692	0.1431	
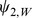	0.0354	5.3513	0.000	
UK				
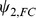	−7.1553	−182.3652	0.0000	*
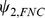	0.1127	4.4023	0.0000	*
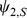	0.0356	3.1587	0.0018	*
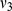	0.0367	1.5176	0.1306	
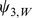	0.0678	5.7961	0.0000	*
IT				
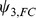	−6.8698	−236.6942	0.0000	*
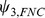	0.0654	2.9420	0.0036	*
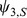	0.0273	2.7861	0.0057	*
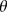	0.0298	1.4161	0.1515	
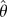	0.0509	5.0062	0.0000	*
**Impact on correlations**				
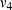	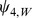	**t-stat**	**p-val**	
US-IT				
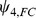	0.6908	42.9045	0.0000	*
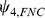	−0.0029	−0.2801	0.6871	
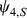	−0.0018	0.3921	0.6954	
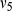	−0.0055	−0.5602	0.5763	
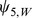	−0.0073	−1.5322	0.1276	
UK-IT				
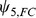	0.8347	77.1971	0.0000	
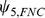	0.0148	2.1081	0.0104	*
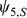	0.0063	2.0330	0.0398	*
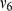	0.0007	0.1091	0.0398	
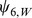	0.0031	0.9532	0.0225	
UK-US				
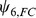	0.7198	38.0473	0.0000	*
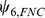	−0.0208	−2.0301	0.0211	*
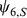	0.0028	0.5631	0.5742	
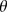	0.0110	1.0191	0.3091	
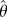	−0.0063	−1.2051	0.2297	

Columns: the parameter 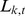 (first), estimates 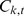 (second), value of the t-statistics (third), p-value of the t-statistics (fourth) and “*” indicates significance of the parameter at the 5% significance level (last).

## Discussion

In an interesting personal account on 2008/2009 events, Peter S. Goodman, now executive business editor of the Huffington Post, past national correspondent for the New Work Times, reported that:

“Inside our newsroom in midtown Manhattan, we understood that were were not merely passive chroniclers of external events. The sportswriter can describe what is happening on the field from a dispassionate distance, without imagining that the words he types may somehow influence the events he is witnessing. Not so for those of us writing about the financial crisis: were were effectively on the field while the game was still under way. Investors and markets and ordinary people would move their money in reaction to what we and other major media were reporting, and this would in turn affect the policy climate, the perception of need for emergency measures, the politics of the debate over those measures, and the public mood, which then reverberated back on everything else” ([63], p. 110).

This personal view testifies to the reflexive nature of markets and the limitation of the mantra of the market efficiency hypothesis. Our findings showed that the idea of reflexivity may contemplate that, in periods of crisis and market unpredictability, even distant information, subjective opinion and economically irrelevant facts may influence market behaviour. Probably, this is due to the fact that today economic, information, technological, and social systems are more strongly and globally coupled than in the past. This means that investors cannot easily predict future outcomes and tend to extend their social reflexivity towards non-operational, non strictly market related events and processes, e.g., by considering that general events, board picture information and the opinion of influential newspapers might have an informative value for markets.

This findings has interesting implications for the financial press. Indeed, following Goodman’s argument, a competent, reliable and responsible information is crucial for markets to work well, especially in periods of financial turmoil. This means that it is important to carry out more serious investigation on the ethics and responsibility of the financial press to establish new standards of conduct and better incentives and sanctions to support reliable information (e.g., [61]; [14]). Secondly, our findings showed that the increasing globalization of financial markets and their correlation in times of crisis require the press to truly cover the international dimension of business and be less parochial. This challenges the quality of the press coverage of global market dynamics and indicates the need for improving the public understanding of the intricate mechanisms of stock markets.

Finally, certain limitations of our work should be considered. First, we did not study the influence of the financial press on stock markets but only that of bad news. This gave us a narrow view of the link between the press and markets. Secondly, we studied the relationship of the financial press and stock markets in an “abnormal” market phase, where market behaviour was strongly subjected to irrational expectation and social mood. We intentionally selected this period as we expected that, in these situations, the pessimism of the financial press and its cross-sectoral dynamics could help us to understand the crisis better. While our results do not contribute to a general theory of the link of press and markets, they can provide important insights to understand the ‘social construction’ of pessimism between press and markets.

Moreover, it is also important to note that press pessimism and market behaviour could also be conditioned by other media, such as the new social media and the Internet (e.g., [31]). Comparing behaviour and impact of various media would be essential to provide a more precise analysis of the 2008/2009 crisis and draw more general remarks of the complex ways through which market sentiment tends to form today (e.g., see [30]). The same holds for the idea of including other newspapers and markets, which may help us to have a more complete picture of the recent crisis and its global dimension.

## Supporting Information

Dataset S1 **Excel file reporting the newspaper data used for the construction of the bad news indexes.**
(XLSX)

Description S1 **Text file with a description of the dataset.**
(TXT)

## References

[1] Reinhart CM, Rogoff KS (2009) This Time is Different. Eight Centuries of Financial Follies. Princeton, NJ: Princeton University Press.

[2] Tickell A (1995) Making a melodrama out of a crisis: reinterpreting the collapse of barings bank. Environment and Planning D: Society and Space 14(1): 5–33.

[3] Thrift NJ (2001) It’s the romance, not the finance, that makes the business worth pursuing: Disclosing a new market culture. Economy and Society 30: 412–432.

[4] Clark GL, Thrift N, Tickell A (2004) Performing finance: The industry, the media and its image. Review of International Political Economy 11(2): 289–310.

[5] Suttles GD, Jacobs MD (2010) Front Page Economics. Chicago: University of Chicago.

[6] Pixley J (2002) Finance organizations, decisions and emotions. British Journal of Sociology 53: 41–65. 11958678 10.1080/00071310120109320

[7] Shiller R (2002) Bubbles, human judgement, and expert opinion. Financial Analysts Journal 58(3): 18–23.

[8] Thaler RH (1993) Advances in Behavioral Finance. New York: Russell Sage Foundation.

[9] Koutmos G (1995) Asymmetric volatility transmission in international stock markets. Journal of International Money and Finance 14(6): 747–762.

[10] Koutmos G (1998) Asymmetries in the conditional mean and the conditional variance: Evidence from nine stock markets. Journal of Economics and Business 50(3): 277–290.

[11] Borges B, Goldstein DG, Ortmann A, Gigerenzer G (1999) Can ignorance beat the stock market? In: Gigerenzer G, Todd PM, the ABC Research Group, editors. Simple heuristics that make us smart, New York: Oxford University Press. pp. 59–72.

[12] Tse Y (1999) Price discovery and volatility spillover in the djia index and futures markets. Journal of Futures Markets 19(8): 911–930.

[13] Soroka S (2006) Good news and bad news: Asymmetric responses to economic information. Journal of Politics 68(2): 372–385.

[14] Kleinnijenhius J, Schultz F, Oegema D, van Atteveldt W (2013) Financial news and market panics in the age of high-frequency sentiment trading algorithms. Journalism 14(2): 271–291.

[15] Veronesi P (1999) Stock market overreaction ti bad bews in good times: A rational expectations equilibrium model. The Review of Financial Studies 12(5): 957–1007.

[16] Beber A, Brandt MW (2010) When it cannt get better of worse: The asymmetric impact of good and bad news on bond returns in expansions and recessions. Review of Finance 14: 119–155.

[17] Entman R (1996) Framing toward clarification of a fractured paradigm. Journal of Communication 43(4): 51–58.

[18] Kahneman DA, Tversky A (2000) Choices, Values, and Frames. Cambridge: Cambridge University Press.

[19] Tan SJ, Chua SH (2004) While stocks last. impact of framing on consumers’ perception of sales promotions. Journal of Consumer Marketing 21(4/5): 343–355.

[20] Severin W, Tankard JW (2005) Communication theories. Origins, Methods, Uses, 5^th^ edition. New York: Addison-Wesley/Longman.

[21] Oberlechner T, Hocking S (2004) Information sources, news, and rumors in financial markets: Insights into the foreing exchange market. Journal of Economic Psychology 25(3): 407–424.

[22] Shiller R (2005) Irrational Exuberance. Princeton, NJ: Princeton University Press.

[23] Knorr Cetina K, Preda A (2005) The Sociology of Financial Markets. Oxford: Oxford University Press.

[24] Antweiler W, Frank MZ (2004) Is all that talk just noise? the information content of internet stock message boards. Journal of Finance 49(3): 1259–1269.

[25] Tetlock PC (2007) Giving content to investor sentiment: The role of media in the stock market. Journal of Finance 62: 1139–1168.

[26] Engelberg JE, Parsons CA (2011) The causal impact of media in financial markets. Journal of Empirical Finance LXVI (1): 67–97.

[27] Preda A (2007) The sociological approach to financial markets. Journal of Economic Surveys 21(3): 506–533.

[28] Schindler M (2007) Rumors in Financial Markets. Insights into Behavioral Finance. Chichester: John Wiley & Sons.

[29] Barber B, Loeffler D (1993) The “dartboard” column: Second-hand information and price pressure. Journal of Financial and Quantitative Analysis 28(2): 273–284.

[30] Preis T, Reith D, Stanley HG (2010) Complex dynamics of our economic life on different scales: insights from search engine query data. Philosophical Transactions of the Royal Society A 368: 5707–5719. 10.1098/rsta.2010.0284 21078644

[31] Bollen J, Mao H, Zeng X (2011) Twitter mood predicts the stock market. Journal of Computational Science 2: 1–8.

[32] Griffin JM, Hirschey NH, Kelly PJ (2011) How important is the financial media in global markets. Review of Financial Studies 24(12): 3941–3992.

[33] Mondria J (2006) Financial contagion and attention allocation. Working Papers 245, University of Toronto, Department of Economics.

[34] Engle R (1982) Autoregressive conditional heteroskedasticity with estimates of the variance of u.k. inflation. Econometrica 50: 987–1008.

[35] Engle R (2002) Dynamic conditional correlation: A simple class of multivariate generalized autoregressive conditional heteroskedasticity models. Journal of Business & Economic Statistics 20(3): 339–350.

[36] Granger CWJ (1969) Investigating causal relations by econometric models and cross-spectral methods. Econometrica 37: 424–439.

[37] Pearce DK, Roley VR (1985) Stock prices and economic news. Journal of Business 58(1): 49–67.

[38] Liu P, Smith SD, Syed A (1990) Stock price reaction to the wall street journal’s securities recommendations. Journal of Financial and Quantitative Analysis 25(3): 399–410.

[39] Tivegna M, Chiofi G (2004) News and Exchange Rate Dynamics. Aldershot: Ashgate.

[40] Stickel SE, Verrecchia RE (1994) Evidence that volume sustains price changes around earnings announcements. Financial Analysts Journal November-December: 57–67.

[41] Balduzzi P, Elton EJ, Green TC (2001) Economic news and bond prices: Evidence from the u.s. treasury market. Journal of Financial and Quantitative Analysis 36(4): 523–543.

[42] Kim SJ, Sheen J (2001) Minute-by-minute dynamics of the Australian bond futures market in response to new macroeconomic information. Journal of Multinational Financial Management 11: 117–137.

[43] Pritamani M, Singal V (2001) Return predictability following large price changes and information releases. Journal of Banking and Finance 25: 631–656.

[44] Brenner M, Pasquariello P, Subrahmanyam M (2009) On the volatility and comovement of U.S. financial markets around macroeconomic news announcements. Journal of Financial and Quantitative Analysis 44(6): 1265–1289.

[45] Tetlock PC (2010) Does public financial news resolve asymmetric information? The Review of Financial Studies 23(9): 3520–3557.

[46] Tetlock PC (2011) All the news that’s fit to reprint: Do investors react to stale information? The Review of Financial Studies 24(5): 1481–1512.

[47] Coval J, Shumway T (2001) Is sound just noise? Journal of Finance 56(5): 1887–1910.

[48] Dougal C, Engelberg J, Garca D, Parsons CA (2012) Journalists and the stock market. The Review of Financial Studies 25 (3): 639–679.

[49] Tetlock PC, Saar-Tsechansky M, Macskassy S (2008) More than words: Quantifying language to measure firms’ fundamentals. Journal of Finance 63: 1438–1467.

[50] Nofsinger JR (2005) Social mood and financial economics. Journal of Behavioral Finance 6 (3): 144–160.

[51] Knorr Cetina K, Bruegger U (2002) Global microstructures: The virtual societies of financial markets. American Journal of Sociology 107(4): 905–950.

[52] Knorr Cetina K, Preda A (2007) The temporalization of financial markets: From network markets to flow markets. Theory, Culture and Society 22: 213–234.

[53] Gentzkow M, Shapiro J (2010) What drives media slant? evidence from U.S. daily newspapers. Econometrica 78: 35–71.

[54] Boero R, Bravo G, Castellani M, Squazzoni F (2010) Why bother with what others tell you? An experimental-data driven agent-based model. Journal of Artificial Societies and Social Simulation 13(3) 6.

[55] Ackerman J (2008) The suprime crisis and its consequences. Journal of Financial Stability 4: 329–337.

[56] Morris S, Hyun SS (1999) Risk management with interdependent choice. Oxford Review of Economic Policy 15(3): 52–62.

[57] Dror Y, Raddant KM, Lux T, Ben-Jacob E (2012) Evolvement of uniformity and volatility in the stressed global financial village. PLoS ONE 7(2): e31144. 22347444 10.1371/journal.pone.0031144 PMC3275621

[58] Cipriani M, Guarini A (2008) Herd behavior and contagion in financial markets. The BE Journal of Theoretical Economics 8(1).

[59] Parsons W (1989) The Power of the Financial Press. Journalism and Economic Opinion in Britain and America. Aldershot: Edward Elgar.

[60] Schifferes S (2011) The financial crisis and the UK media. In: Schiffrin A, editor. Bad News. How America’s Business Press Missed the Story of the Century. New York: The New Press. pp. 148–178.

[61] Tambini D (2010) What are financial journalists for? Journalism studies 11 (2): 158–174.

[62] Doyle G (2006) Financial news journalism. Journalism 7(4): 433–452.

[63] Goodman PS (2011) The quiet crisis. In: Schiffrin A, editor, Bad News. How America’s Business Press Missed the Story of the Century. New York: The New Press. pp. 94–121.

[64] Engle RF, Ng VK (1993) Measuring and testing the impact of news on volatility. Journal of Finance 48(5): 1749–1778.

[65] Forbes K, Rigobon R (2002) No contagion, only interdependence: measuring stock market comovements. Journal of Finance 57 (5): 2223–2261.

[66] Chiang TC, Jeon BN, Li H (2007) Dynamic correlation analysis of financial contagions: Evidence from asian markets. Journal of Internatonal Money and Finance 26: 1206–1228.

[67] Longin F, Solnik B (2001) Extreme correlation of international equity markets. Journal of Finance 56 (2): 649–676.

[68] Jondeau E, Rockinger M (2006) The copula-garch model of conditional dependencies: An international stockmarket application. Journal of International Money and Finance 25 (5): 827–853.

