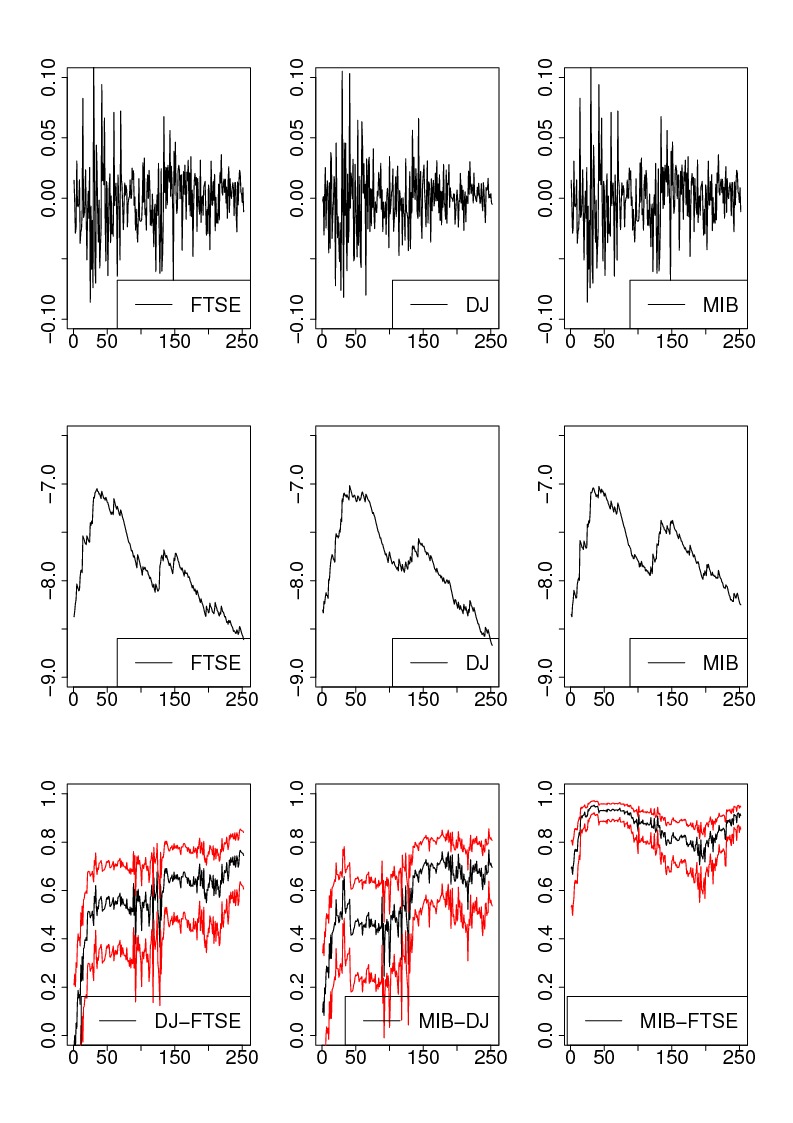# Correction: Being on the Field When the Game Is Still Under Way. The Financial Press and Stock Markets in Times of Crisis

**DOI:** 10.1371/annotation/97adcadf-bf3b-45f0-af5a-9133a7a638d0

**Published:** 2014-01-17

**Authors:** Roberto Casarin, Flaminio Squazzoni

The affiliation for the last author was incorrect. Flaminio Squazzoni is affiliated with: Department of Economics and Management, University of Brescia, Brescia, Italy.

In Table 7 and Table 8, the Greek letters indicating the parameters are missing.

Please see the corrected Table 7 here: 

**Figure:**
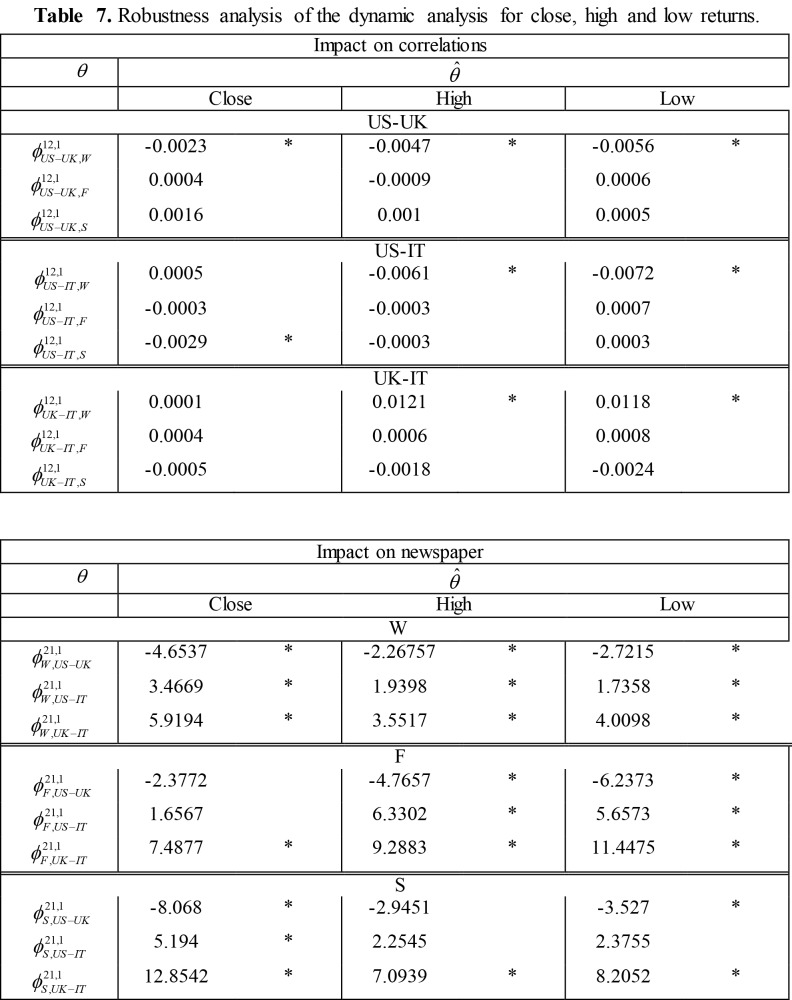


Please see the corrected Table 8 here: 

**Figure:**
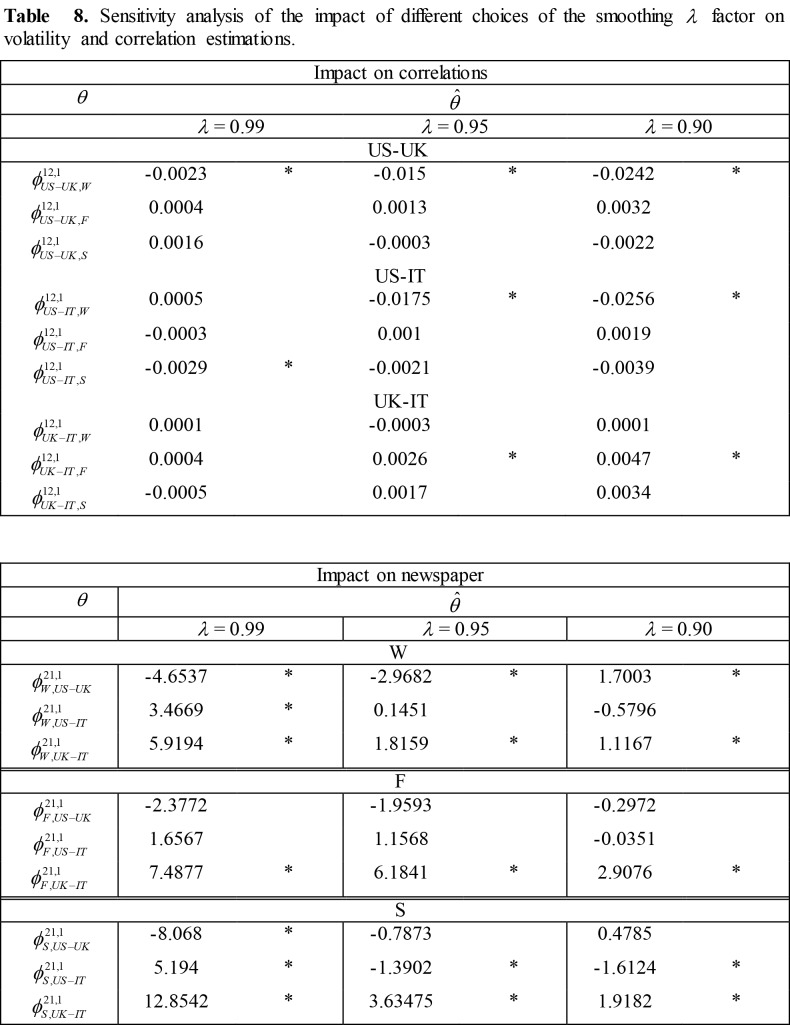


Figure 2 has been reformatted for better readability. Please see Figure 2 here: 

**Figure:**